# Field Evidence of Colonisation by Holm Oak, at the Northern Margin of Its Distribution Range, during the Anthropocene Period

**DOI:** 10.1371/journal.pone.0080443

**Published:** 2013-11-18

**Authors:** Sylvain Delzon, Morgane Urli, Jean-Charles Samalens, Jean-Baptiste Lamy, Heike Lischke, Fabrice Sin, Niklaus E. Zimmermann, Annabel J. Porté

**Affiliations:** 1 INRA, UMR 1202 BIOGECO, Cestas, France; 2 Université de Bordeaux, UMR 1202 BIOGECO, Cestas, France; 3 INRA, UR 1263 EPHYSE, Villenave d'Ornon, France; 4 Swiss Federal Research Institute WSL, Birmensdorf, Switzerland; 5 ONF, Agence de Bordeaux, Bruges, France; Lakehead University, Canada

## Abstract

A major unknown in the context of current climate change is the extent to which populations of slowly migrating species, such as trees, will track shifting climates. Niche modelling generally predicts substantial northward shifts of suitable habitats. There is therefore an urgent need for field-based forest observations to corroborate these extensive model simulations. We used forest inventory data providing presence/absence information from just over a century (1880–2010) for a Mediterranean species (*Quercus ilex*) in forests located at the northern edge of its distribution. The main goals of the study were (i) to investigate whether this species has actually spread into new areas during the Anthropocene period and (ii) to provide a direct estimation of tree migration rate. We show that *Q. ilex* has colonised substantial new areas over the last century. However, the maximum rate of colonisation by this species (22 to 57 m/year) was much slower than predicted by the models and necessary to follow changes in habitat suitability since 1880. Our results suggest that the rates of tree dispersion and establishment may also be too low to track shifts in bioclimatic envelopes in the future. The inclusion of contemporary, rather than historical, migration rates into models should improve our understanding of the response of species to climate change.

## Introduction

Global temperature rises, increasing atmospheric CO_2_ concentration, nitrogen deposition, changes in land use and forest management have altered the production and biodiversity of the terrestrial biosphere [1], [2]. Concerns have been raised about the responses to ongoing climatic change in tree species, particularly given the rapid rate of environmental change and the long life-span of these species [3]. Trees may adopt various strategies (migration, adaptation) to cope with rapid environmental changes and landscape fragmentation, but adaptation alone (through genetic adaptation and phenotypic plasticity) is unlikely to be rapid enough to maintain standing populations [4]. A combination of migration and adaptation is, therefore, likely to be the only solution permitting the sustainable survival of tree populations. As a consequence, the boundaries of species distribution ranges may shift. Recent changes in plant distributions have, indeed, been reported in recent decades [1], [5]. These migrations are however evident in mountain areas only, in which plant populations are mostly shifting upwards [6]–[12]. Studies of tree responses to rapid changes in climate are thus essential, to provide us with insight into the possible future distributions of these species and biodiversity.

A better understanding of the link between climate change and distribution range dynamics can be obtained from studies of past changes. Phylogeographic, palynologic and anthracological studies have quantified shifts in tree distribution ranges due to past climate change (alternations of glacial and post-glacial warming periods), providing essential information for the determination of migration directions and rates and for forecasting future shifts of tree species distribution ranges [13], [14]. Fossil pollen data have suggested that northward migration is typical for many tree species responding to post-glacial climate warming [15], [16]. The ranges of many temperate tree species were estimated to have expanded at rates of 500–1000 m year^−1^ during the early Holocene [17], but these palynological studies showed that migration rates slow considerably after an initial phase of rapid invasion [17]. One well documented example of range shift is that for Holm oak (*Quercus ilex*), which displayed westward colonisation in the Mediterranean Basin during the Tertiary, demonstrated by studies of chloroplast DNA variation [18]. It remains unclear whether past migrations of trees were limited by rates of climate change or by their dispersal abilities [19], but past rates of migration suggest that even relatively rapid changes in range limits would be insufficient to keep pace with predicted future climatic changes [20], [21].

Vegetation distribution models provide additional insight, improving our understanding of the impact of climate change on species distribution and biodiversity. Niche-based models accounting for observed geographical distribution ranges on the basis of climatic and other environmental variables are frequently used to forecast the potential impact of ongoing climatic changes on the distribution and size of plant and animal ranges [22]–[26]. Many studies based on the use of such models have suggested that species distributions may shift in response to climate change (mostly towards higher latitudes and altitudes; e.g. [27], [28]). In the future, climatic conditions may be such that the magnitude of potential range changes is large even for trees [29]. Iverson and Prasad [30] evaluated potential changes in suitable habitats for tree species in the eastern United States and showed a substantial northward shift for 60 of the 80 species studied. Using eight models, ranging from niche-based models to process-based models including ecophysiological processes but no structural dynamics or seed dispersal, Cheaib *et al.* [31] predicted a significant range expansion, by 2055, for Mediterranean broad-leaved evergreen species (mostly Holm oak) over the western-most two thirds of France. All models have predicted massive range expansions for this species, but they yield very different predictions concerning the magnitude and location of the changes. Thus, although there is sufficient space and suitable habitats for the northward expansion of the distribution ranges of many species, the degree to which species are actually able to achieve such rapid migration remains poorly understood (see [32] for review): model uncertainties concerning the rate and direction of migration create broad differences in projections of the impact of climate change on tree species ranges [31], [33]. Thus, in addition to more explicit modelling of the processes underlying migration [34], there is a need for field-based forest observations to confirm these extensive model simulations. Appropriate field observation data for the derivation of migration rates are scarce but of great importance, if we are to understand the response of tree species to global changes [32].

We investigated changes in the distribution of a Mediterranean tree species (*Quercus ilex*) at the northern edge of its range. This species is expected to be strongly affected by ongoing climate change [31]. Our main purpose was to check the veracity of the recent forecasts of changes in distribution area for this species. We focused on the situation in western France, an area corresponding to the northern limit of the distribution range for this Mediterranean oak. We used field-based forest inventories from the French National Forestry Office, providing presence/absence information for a period of just over a century (1880–2010) for *Quercus ilex* in four state-owned forests. The two specific goals of the study were (i) to check whether this Mediterranean species is currently colonising new sites at its leading edge, with a magnitude similar to that predicted by models and (ii) to provide a direct estimate of the rate of colonisation from field observations. By quantifying contemporary changes in tree distribution based on reliable information, this study will improve our understanding of the extent to which species are likely to move in the future.

## Materials and Methods

### Study area and climate

**Figure 1:**
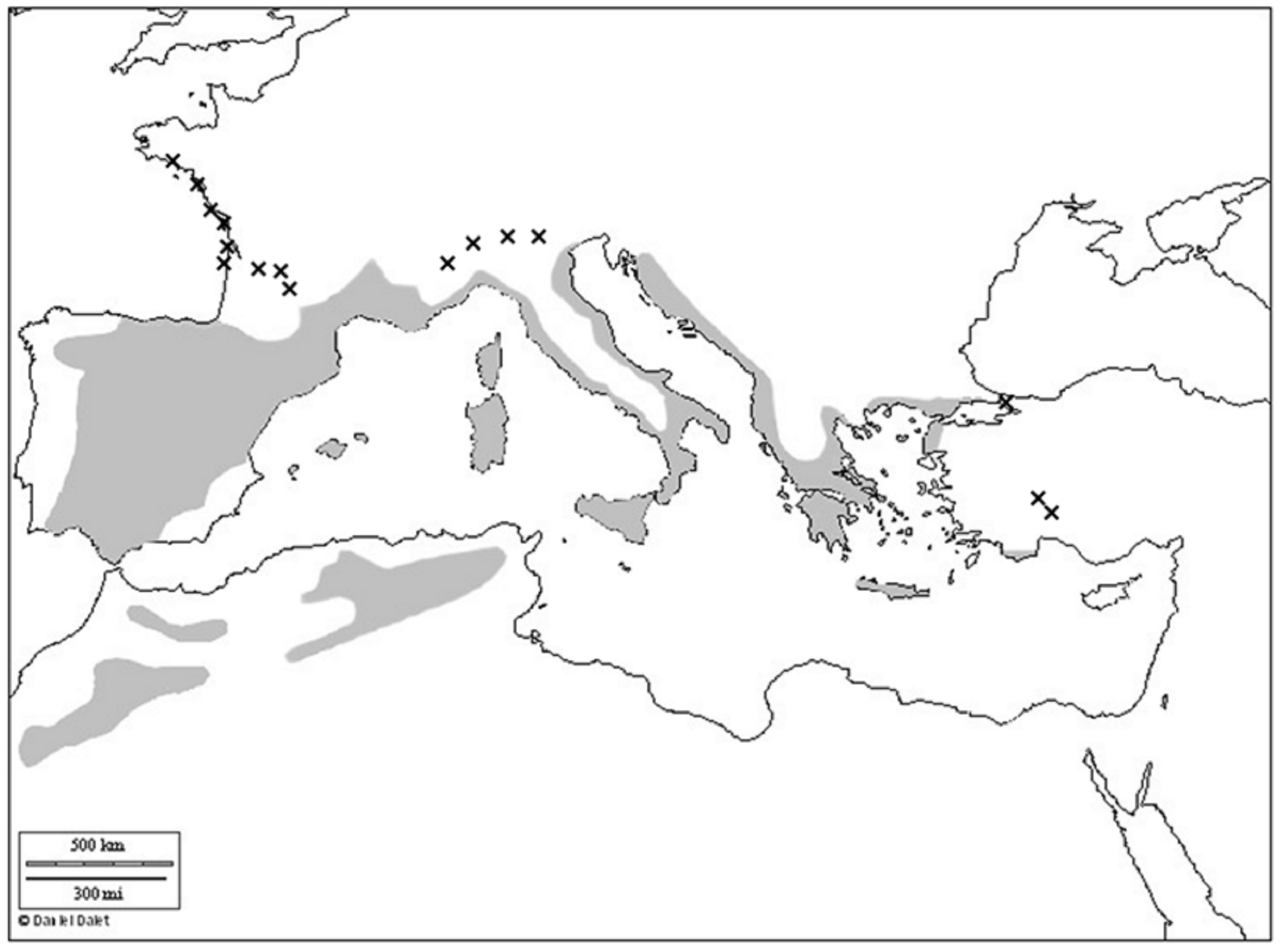
Map of the current distribution range of Holm oak (*Quercus ilex*) in the Mediterranean basin. The distribution range of *Quercus ilex* is shown in grey and crosses represent geographically isolated populations. The map was established according to the findings of Barbero *et al*. [74], Michaud *et al.* [75] and raw data from the French National Forest Inventory (http://inventaire-forestier.ign.fr/edb/query/show-query-form#consultation_panel). The blank base map was provided free of charge and use by Daniel Dalet/Académie Aix-Marseille (http://www.ac-aix-marseille.fr/pedagogie/jcms/c_67064/fr/cartotheque).

This study was conducted at the northern edge of the distribution range of Holm oak (*Quercus ilex* L.) in Europe, along the French Atlantic coast, from Bordeaux to Nantes (figure 1). This evergreen tree of the Fagaceae family is a typical component of the flora in Mediterranean climates, its largest populations extending throughout the Iberian Peninsula. The Holm oak populations of the Atlantic coast dunes in France constitute the northern-most occurrences of Mediterranean evergreen trees. *Q. ilex* is considered to be drought-tolerant [35], [36]. It displays tight stomatal control over transpiration, but may nevertheless display embolism and a loss of hydraulic conductance under severe drought stress [37], [38]. The major limitations to the persistence of Holm oak at its northern margin are damage to photosynthetic capacity [39] and cold-related damage to young plants [40]. Holm oak acorns are dispersed during fall mainly by rodents and European jays (*Garrulus glandarius*). They usually transport acorns far from adult oaks [41], [42]. The average distance of acorn dispersal is around 250 m, with dispersal events occurring up to 1 km from source Oak trees. Usually age at maturity of resprouter individuals is 10 years and acorn size can vary strongly between years and sites, with the biggest acorns preferably consumed by predators (wild boars), which might hamper regeneration.

**Figure 2:**
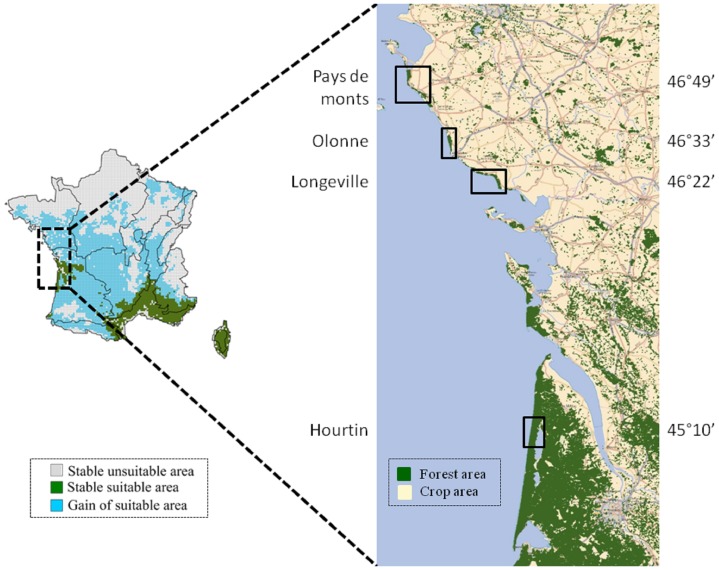
Map of the study area, with locations and mean latitudes of the four state-owned forests used in the retrospective analysis (Pays de Monts, Olonne, Longeville, and Hourtin; right panel). These studied forests represent the northern limit of Holm oak distribution in Europe. All forest covers are represented in green colour. The map in the left panel shows the changes in Holm oak suitable habitat by 2055 in France as predicted by the BIOMOD niche-based model [31]. The base map featuring the forested areas was extracted free of charge and use from the IGN National Forest Inventory cartography site ((© IGN 2013 – BD Forêt V1 des départements 33,17, 85 and FranceRaster® V.1 (2007 ©IGN/CARTOSPHERE))).

We carried out a retrospective analysis of four state-owned forests covering a broad range of Atlantic dune forests; these forests have been managed by the National Forestry Office (ONF) since 1880 (figure 2). They are mixed pine-oak forests composed principally of a maritime pine (*Pinus pinaster*) overstorey and a codominant or dominant Holm oak (*Quercus ilex*) canopy, interspersed with scarce, sparse patches of pedunculate oak (*Quercus robur*) at the base of the dunes. These Atlantic coastal dune forests (maximum canopy height of 60 m) are subject to a maritime climate, with a mean annual temperature of 12.5°C and precipitation of 850 mm (see table 1). The soil is an arenosol with a filtering siliceous sandy texture and low organic matter content, varying little between sites. The climate warming recorded in France in the 20th century is about 30% greater than mean global warming levels. It has been particularly marked in south-western France, where temperatures have risen by 1.1°C [43] mostly during the second half of the 20th century (1.5°C from 1960 to 2000 in the studied areas, figure S1). This temperature increase has been accompanied by an increase in precipitation during the autumn and winter and lower levels of precipitation in the summer, exacerbating the summer drought period [43].

**Table 1:** Location and climatic characteristics of the four forests studied.

Forest	Areaha	Latitude	Longitude	MAPmm	MAT°C
Pays de Monts	2009	46°49’ N	2°08’ W	897	12.11
Olonne	1106	46°33’ N	1°49’ W	821	12.20
Longeville	1520	46°22’ N	1°28’ W	772	12.53
Hourtin	3621	45°10’ N	1°09’ W	864	12.93

Climate data were obtained from Meteo France. MAP is the mean annual precipitation over the period 1961-1990; MAT is the mean annual temperature over the period 1961-1990.

### Retrospective survey data

For more than 120 years, the French National Forestry Office (ONF) has managed the state-owned forests along the Atlantic coast, for which it has carried out a comprehensive inventory of the species present. Four to seven surveys have been carried out during this extensive period, depending on the forest considered. However, in all the forests studied, the first survey was conducted between 1880 and 1891, and the last survey was carried out around the start of the 21^st^ century. All inventories were carried out according to the same methodology in all plots (15 to 90) depending on the studied forest). Briefly, forest managers investigated forest plots, scoring various types of tree species (broadleaved oaks, evergreen oak, pines) as present or absent (including seedlings, saplings and adult trees without distinction). As Holm oak is the only evergreen oak growing in these forests, its presence/absence within all plots was known with certainty. Forest-covered plots were explored at least once during each of the survey periods, such that geographic coverage could be considered complete and identical for all surveys. The observed presence of this species in these forest plots was then used for the construction of maps. The distribution maps obtained during different periods therefore provide estimates of changes in the area occupied by Holm oak.

The range of a colonising species spreads to become irregular in shape over geographic space, due to stochastic processes, geographic boundaries and multiple colonisation fronts. We therefore estimated the colonisation rate based on the colonized area rather than using a unidirectional distance, which might result in overestimation. For each inventory date, the colonised area, *a*, was measured by summing the areas of plots in which *Q. ilex* was observed. Assuming an omnidirectional spread of this species within each studied forest, the radius of a circle of the same area was calculated. The rate of colonisation between two inventories (*t*_+1_, *t*) was then estimated as follows:
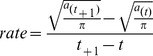



The maximum colonisation rate for each forest studied (highest values throughout the entire period) was preferably used in order to (i) determine the capacity of tree populations to migrate, and (ii) avoid bias due to spatial constraints related to the forest boundaries. Thus we did not compare mean values of colonization rate during the studied period because recent rates were constrained by the limited size of the forests.

## Results and Discussion

**Figure 3:**
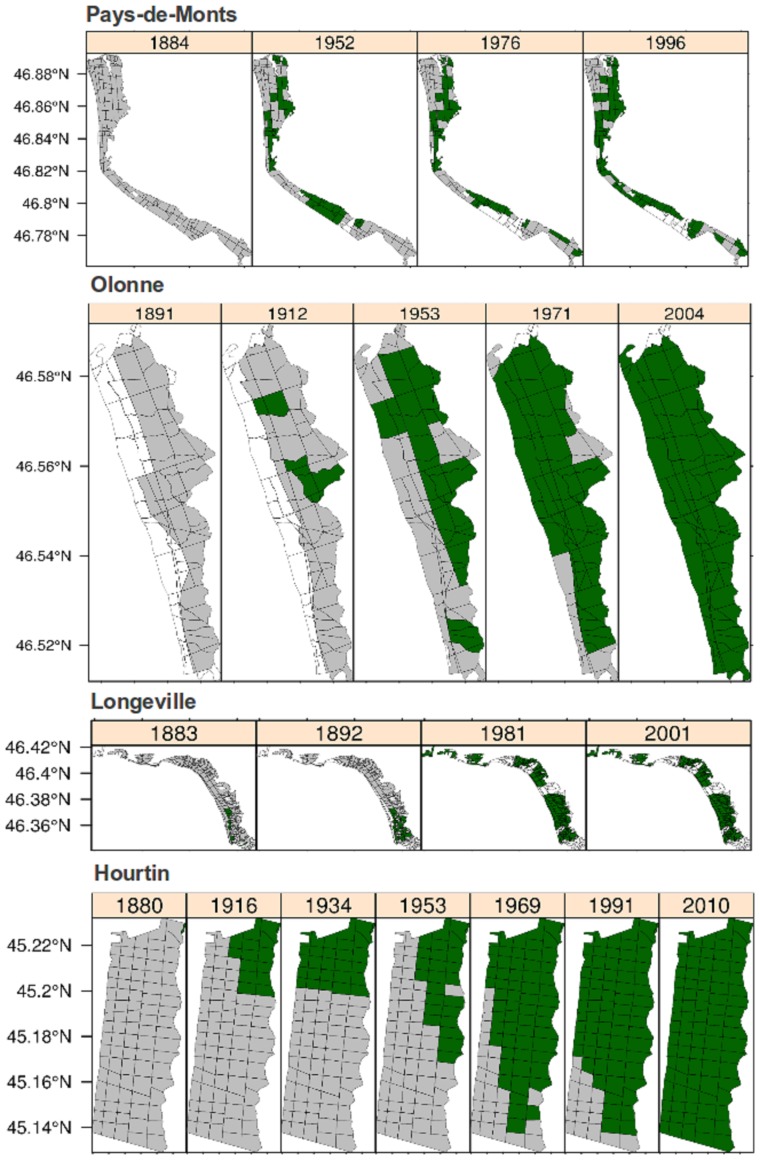
Changes in the area of Holm oak over the 20^th^ century in each of the forests studied. The presence of Holm oak is indicated in green, the forest area is indicated in grey and the non-forest area is indicated in white. Forests are ordered by latitude, from bottom to top: Hourtin, Longeville, Olonne and Pays de Monts. Please note the different scales used.

We found that the distribution of *Q. ilex* had changed markedly over the last century, with massive colonisation occurring in all the studied forests along the Atlantic coast (figure 3). From relict individuals in the studied forests, this species began to colonise new areas in the early 1900s, spreading considerably in the middle of the 20^th^ century. Most inventories now indicate the presence of Holm oak throughout the entire area of the forests investigated. There have been only a few detailed case studies of range expansion in trees during the Anthropocene period, although such events may be very common. Indeed, long-term presence/absence data based on field observations are very scarce. Walther *et al*. [44] demonstrated range expansion for *Ilex aquifolium* in northern Europe in association with an increase in winter temperatures. Forest inventories have been used to estimate tree migration, but always in an indirect manner, by comparing the latitude for seedling and tree biomass [45], or by comparing latitudinal distributions of abundance and occupancy [46], for example. Such studies have concluded that most tree species are currently colonising new areas at their northern margins in the eastern USA. However, these studies have tended to analyse dynamics at the core of the distribution, rather than at its limits. Their analyses therefore fail to portray the dynamics at the northern margin of the species concerned [45]. Zhu *et al*. [47] recently reported that there was little evidence of climate-induced tree migration, with only a few species (20%) presenting a pattern consistent with a northward shift of their northern distribution margin. In a similar study, Gehrig-Fasel *et al*. [48] found that only a small proportion (5%) of observed shift close to or at the upper tree line in Switzerland could readily be attributed to climate-induced migration, the remaining observed changes instead reflecting changes in land use. In the same way, the treeline of *Abies alba* has shifted upward in the Alps during the second half of the 20^th^ century, mainly in response to land use change, even though the role of climatic warming cannot be ruled out [49].

**Figure 4:**
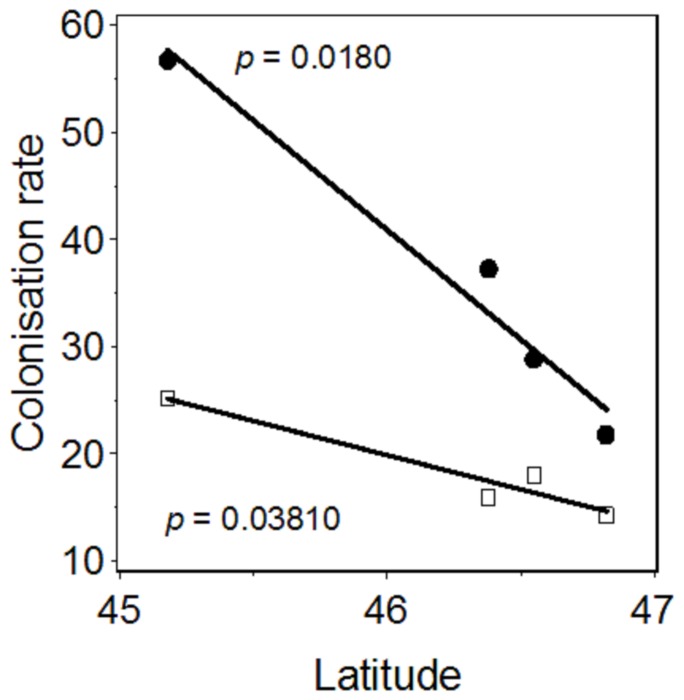
Colonisation rates (m year^−1^) as a function of the latitude (°) of the studied forests. Maximum (closed circle) and mean (open square) values were estimated for each forest. The *P*-values indicated are those for linear regression analysis.

The data presented in table 2 are derived from the colonisation maps generated over periods of 112 to 130 years, for similar local spatial scales in each case. The highest values of colonisation rates for the forests studied were between 22 and 57 m yr^−1^. Colonisation rates were fastest in the southern-most forest of the study area (Hourtin) and decreased with increasing latitude (figure 4), but the changes observed were of similar magnitude in all forests over the period studied. The estimated colonisation rates were much lower than those estimated for post-glacial migrations of trees (> 500 m year^−1^ [1], [50], [51]), probably because colonising individuals do not encounter established forests during the Holocene [17]. Competition with other species at a local scale may thus have slowed Holm oak colonisation during the Anthropocene [34]. The potential of a species to colonise a new area inevitably depends on its performance relative to those of the resident species, and interactions with other species may clearly slow establishment and maturation, thereby decreasing the rate of migration of a tree species, thereby decreasing the capacity of that species to track the changing climate [52]. The difference in colonisation rates between the Holocene and Anthropocene periods may also reflect habitat fragmentation due to human activity [53], [54], forest management or, simply, an overestimation of past migration rates in cases in which outlier populations invisible to the pollen record nucleate range expansions [55]. Indeed, some recent studies have estimated rates of tree species migration during the Holocene of only one fifth to one tenth (<100 m year^−1^) previously reported values. Such discrepancies may be due to the presence of late glacial refugia close to the northern limits, and a lack of knowledge of these refugia may have biased earlier estimates [56], [57]. Our estimated rates (<60 m year^−1^) are consistent with model predictions based on population dynamics, competition and dispersal [58], [59], seed dispersal data (12.5 m year^−1^ for *Q. robur* and *Q. petraea* [60]) and cell-based migration simulation models (probability of colonisation within a zone of 10–20 km around the area currently occupied [61]).

**Table 2:** Colonisation rates (m year^−1^) for Holm oak, estimated by the analysis of its presence distribution maps in the four studied forests along the latitudinal gradient.

Hourtin		Longeville		Olonne		Pays de Monts	
year	m yr^−1^	year	m yr^−1^	year	m yr^−1^	year	m yr^−1^
1880–1916	36.5	1883–1892	37.3	1891–1912	28.9	1884–1952	21.8
1916–1934	21.0	1892–1981	10.5	1912–1953	17.5	1952–1976	3.9
1934–1953	5.0			1953–1971	20.4	1976–1996	17.3
1953–1969	56.7			1971–2004	5.2		
1969–1991	6.8						
1991–2010	23.1						
**maximum rates**	**56.7**		**37.3**		**28.9**		**21.8**

By implicitly including biotic interactions between species, our migration rate estimates represent the actual capacity of the species to disperse into existing forests. They are, therefore, more representative when assessing the response of trees to current climate change than migration rate estimates from paleoecological studies of the last glaciation–deglaciation cycle. However, our field observations were made at a local spatial scale (<4000 ha), so an underestimation of migration rates cannot be excluded. Indeed, the importance of long-distance dispersal for migration has been demonstrated theoretically [62], [63]. Seed dispersal is usually considered to be long-distance if it occurs over a distance of more than 100 m. Our study therefore excluded only rare, very long-distance dispersal events (>5 km). However, Iverson *et al.* [61] demonstrated that a species has to have a reasonable abundance close to the limit of its range for colonisation to be likely. These authors concluded that very long-distance migration events alone might not be sufficient to rescue migration. The decreasing colonisation rate with increasing latitude found here confirms the importance of population abundance in the process of migration, with much lower rates of colonisation occurring in the northern-most populations (Pays de Mont/Olonne exhibit the same temperature change over the period studied but dramatically lower abundance of Holm oak).

Our findings confirm that this Mediterranean species is colonising new areas at the northern limits of its distribution range and that it therefore has the potential to shift the edge of its range northwards under projected climate change, as predicted by both niche-based and process-based models [31]. Indeed, all the various types of model indicate that the climate will probably become favourable for this species well to the north of its current bioclimatic range. However, we show here that despite the clear occurrence of migration in this tree species, colonisation rates are much lower than predicted by models. The estimated migration rates obtained here are two orders of magnitude below those required to track climate change. Indeed, estimates of the migration rates required for plant species to keep pace with climate change over the coming century are frequently >1000 m year^−1^ [64], [65], exceeding even the fastest migration rates observed during early Holocene colonisation [66]. However, none of these models takes dispersal capacity into account [31], and their projections of increases in range size should therefore be interpreted with caution. Only a few models have included specific migration rates, estimated from data obtained from paleoecological studies of the last glaciation–deglaciation cycle. The rates used in these models were thus between 1000 and 10000 m year^−1^, depending on the species considered [67]. Sensitivity analyses of these models have generally identified migration rate as a crucial issue, and their results have indicated that colonisation remains strongly limited by migration rates, even if a rate of 1000 m year^−1^ is used [65]. Finally, our results clearly confirm that the migration of the species studied here will be limited by its ability to disperse and to colonise habitats that have recently become suitable. The time lag for species establishment in new areas is unknown, and similar field estimates of migration rates should be used and combined with modelling approaches, to improve the accuracy of projections and inferences concerning the future distributions of tree species.

This range expansion observed at the northern margin of the Holm oak distribution is likely explained by both climate and recent management changes but their relative contribution cannot be disentangled. Across the region studied, mean annual temperatures have rapidly increased, by 1.5°C during the 20^th^ century, leading to more arid conditions, due to higher evapotranspiration rates and a change in the seasonal pattern of annual precipitation [68]. This climate warming is probably one of the key drivers of the observed range expansion of Holm oak over the last century, as suggested by the relationship between colonisation rates and latitude or mean annual temperature (MAT; table 1, figure 4). Northern range limitation by temperature has commonly been reported for various plant species [44], [69], [70]. On the other hand, during the first half of the 20^th^ century (slight change in temperature; see figure S1), forest management probably initially moderated colonisation by Holm oak, limiting the expansion of the range of this species, because there was a general policy of cutting down Holm oaks to favour monospecific maritime pine stands [71], [72]. However, this systematic felling policy ceased after the 1950s, when forest managers began to support natural forest dynamics, including Holm oak colonisation. This change in forestry practices has strongly influenced the recruitment of *Quercus ilex* and therefore its colonization rate. However, in all the forests studied, no land abandonment or Holm oak plantation has been observed; thus the pattern of colonization reported here cannot be attributed to land-use change while in contrary it played a major role in mountainous areas [49]. Both these successive changes in management and the sharp increase in temperature during the second half of the 20^th^ century may have led to an increase in the rate of expansion of the distribution of Holm oak, as this species is known to recover rapidly from disturbances, such as felling and drought, through highly dynamic resprouting [73]. These changes were indeed reflected in estimated colonisation rates, which were higher in the second half of the 20^th^ century (table 2).

## Conclusions

The area along the Atlantic coast of France colonised by Holm oak increased steadily throughout most of the 20^th^ century due to both management and climate changes, but the rate of range expansion by migration is much lower than would be required to track future climate change according to the predictions of niche modelling and phylogeographic studies. At its current rate of expansion, this species will not be able to colonise all the climatically suitable habitats that are likely to appear in France and elsewhere over the next 50 years or so. Even if a population disperses to a new region with a favourable climate, interactions with other species may prevent its establishment and further spread. Thus, predictions of substantial range shifts should probably be tempered, at least for plant species, and particularly for long-lived species, such as trees. The prediction of shifts in species ranges as a result of climate change could be improved by taking migration processes into account explicitly in models. Measurements of contemporary colonisation rates are invaluable for providing realistic estimates of population range dynamics, and should be obtained and used more frequently in assessments of the potential of a species to track climate change.

## Supporting Information

Figure S1 Temperature trends in the four studied areas over the twentieth century (left) and over the four last decades (right). Linear regressions fitted to the annual means are also depicted and showed an increase of 1.5°C during the last four decades. Temperature changes were statistically significant at *P*<0.001 level and no significant differences of temperature changes have been found between areas. Data sources: Météo France (stations n° 33009001, 33236002, 85216001 and 85234001).
(DOCX)
