# Supplementary material for: Field Evidence of Colonisation by Holm Oak, at the Northern Margin of Its Distribution Range, during the Anthropocene Period
Source: PLoS One. 2013 Nov 18;8(11):e80443. doi: 10.1371/journal.pone.0080443 (PMC3832392; doi:10.1371/journal.pone.0080443)
Supplement: Figure S1 — Temperature trends in the four studied areas over the twentieth century (left) and over the four last decades (right). Linear regressions fitted to the annual means are also depicted and showed an increase of 1.5°C during the last four decades. Temperature changes were statistically significant at P<0.001 level and no significant differences of temperature changes have been found between areas. Data sources: Météo France (stations n° 33009001, 33236002, 85216001 and 85234001). (DOCX) [file pone.0080443.s001.docx]

**SUPPLEMENTARY INFORMATION**

**Figure S1.** Temperature trends in the four studied areas over the twentieth century (left) and over the four last decades (right). Linear regressions fitted to the annual means are also depicted and showed an increase of 1.5°C during the last four decades. Temperature changes were statistically significant at *P*<0.001 level and no significant differences of temperature changes have been found between areas. Data sources: Météo France (stations n° 33009001, 33236002, 85216001 and 85234001).
